# Use of Different Organic Carbon Sources in *Cynara cardunculus* Cells: Effects on Biomass Productivity and Secondary Metabolites

**DOI:** 10.3390/plants11050701

**Published:** 2022-03-05

**Authors:** Maria Oliviero, Antonio Luca Langellotti, Giovanni L. Russo, Marco Baselice, Andrea Donadio, Alberto Ritieni, Giulia Graziani, Paolo Masi

**Affiliations:** 1 CAISIAL Center, University of Naples Federico II, 80055 Portici, Italy; maria.oliviero2@unina.it (M.O.); marco.baselice@unina.it (M.B.); andrea.donadio992@gmail.com (A.D.); masi@unina.it (P.M.); 2 Unit of Food Science and Technology, Department of Agricultural Sciences, University of Naples Federico II, 80055 Portici, Italy; giovanniluca.russo@unina.it; 3 Department of Pharmacy, University of Naples Federico II, 80131 Naples, Italy; ritialb@unina.it (A.R.); giulia.graziani@unina.it (G.G.)

**Keywords:** cardoon, sugars, polyphenols, fatty acids, cell culture

## Abstract

*Cynara cardunculus* (Asteraceae family) is a perennial plant native to Mediterranean regions. This plant represents a source of high-value compounds, such as polyphenols and fatty acids that have several industrial applications. However, in vitro plant cell cultures can represent a valid alternative to in-field cultivation and facilitate the extraction of metabolites of commercial interest. Generally, sucrose is the main sugar used for plant cell cultures, but other carbon sources can be considered. Here, we investigated the potential use of alternative organic carbon sources, such as galactose, maltose, glucose, glycerol, fructose, lactose, and starch, for the cultivation of *C. cardunculus* cells. Moreover, cardoon cells were collected, and an extraction of polyphenols and oils was performed to study the effects of different carbon sources on the production of bioactive molecules. This study provided evidence that cardoon cell growth can be supported by carbon sources other than sucrose. However, the carbon source inducing optimum growth, did not necessarily induce the highest production of high-value compounds.

## 1. Introduction

*Cynara cardunculus* (common name, cardoon) is a perennial plant of Asteraceae family, native to Mediterranean areas. The plant, characterized by a high biomass and secondary metabolites production, is cultivated for industrial purposes due to the versatility of the cardoon biomass that makes this crop a promising raw material for biorefineries [1]. The whole plant can be used for medicinal purposes, as well as in the food industry as rennet for cheese production [2,3]. Moreover, the biomass can be used to produce solid biofuel from lignocellulosic biomass, biodiesel from oil and different bio-products like bioplastic, food fragrances, home, and personal care items [1]. *C. cardunculus* is an important source of bioactive compounds that can find several applications in pharmaceutical and nutraceutical industries [4]. Cardoon plant, in fact, is rich in phenolic compounds, mainly mono- and dicaffeoylquinic acids (like cynarin) and flavonoids, which are widely used for their antioxidant activity in pharmacology [2,4]. Cardoon seeds are rich in lipids useful for biofuel production and human consumption [2,5,6]. The oil is characterized by a high nutritional value due to the high amounts of unsaturated fatty acids (linoleic and oleic acids) and low saturated fatty acids amounts (palmitic and stearic acids) [7]. 

Over the years there has been a development of plant cell culture techniques due to the commercial and industrial interest for secondary metabolites and high added value molecules obtained from plants [8]. Plant tissue cultivation is the most prominent plant biotechnology technique, representing an important source of primary and secondary metabolites [9]. These techniques have shown several advantages over the conventional cultivation of whole plant (field-grown crop) because, in this way, the production of secondary metabolites is made under artificial conditions, therefore, leading to results independent of climatic factors. Moreover, the isolation of high added value molecules is more rapid with respect to the extraction from whole plants [8]. The use of plants callus for industrial applications is widespread [10] and, for *C. cardunculus* the induction of callus and its in vitro culture have been widely described [11,12,13]. In particular, *C. cardunculus* leaves represent the most suitable explants for tissue culture in vitro because they are less prone to necrosis [14,15].

Heterotrophic cell suspensions require an exogenous carbon source to grow. For plant cell cultures, several mono- and disaccharides (such as sucrose, glucose, fructose, lactose, and others), have been studied as potential sources of organic carbon able of supporting cellular growth. However, sucrose is the most used organic carbon source [16,17], due to its high water solubility, electrical neutrality, and apparent lack of inhibitory effect when used at standard concentration [18]. Nevertheless, it is important to note that an optimal carbon source for cell growth does not necessarily results in optimal production of high value-added compounds [16]. 

The ability of carbon sources to support the growth of cellular suspensions is also species specific [16,18]. Among sucrose, glucose, and fructose, sucrose is the carbon source that most stimulates the growth and rosmarinic acid accumulation in suspension cultures of *Coleus blumei* [19]. A study on the effect of different sugars in *Silene vulgaris* callus showed that glucose, galactose, and sucrose positively affect polysaccharides biosynthesis [20]. Mello et al. [18] tested, for three different plant cell suspensions, the utilization of glycerol, sorbitol, and galactose as carbon sources alternative to sucrose, reporting that sucrose represents the best carbon source for all the three species tested. Cell cultures of *Rubus arcticus*, instead, were tested for the ability to use lactose as complete substitute for sucrose, but the results reported a significant biomass reduction [21]. 

To the best of our knowledge, no studies were found in literature about the effects of different carbon sources on *C. cardunculus* cells, therefore, the aim of this work was to investigate the capability of cardoon cell suspensions to use eight organic carbon sources, with the consequent effects on biomass productivity and production of secondary metabolites and fatty acids. 

## 2. Results

### 2.1. Optimization of Growth Conditions for Cynara cardunculus In Vitro Cell Cultures

**Figure 1:**
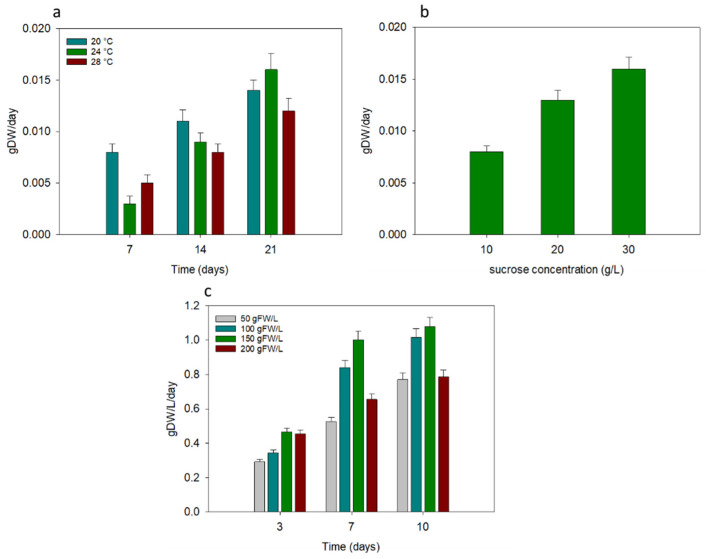
Optimization of growth conditions for in vitro cardoon cells by testing several temperatures, sugar concentrations, and inoculum size. (**a**) Callus cultures tested at three different temperatures such as 20, 24, and 28 °C; (**b**) callus cultures tested at three different sucrose concentrations such as 10, 20, and 30 g L^−1^; (**c**) cellular suspensions tested at four different inoculum sizes, i.e., different initial cellular concentration, such as 50, 100, 150, and 200 g FW (fresh weight) L^−1^.

Growth conditions of *C. cardunculus* cells were optimized and the results are reported in Figure 1.

In Figure 1a the effects of different temperatures on callus cultures are shown: after 21 days, the highest biomass productivity was observed for callus growth at 24 °C (0.016 g DW day^−1^), while at 28 °C the lowest biomass productivity was reported (0.011 gDW day^−1^). Therefore, at an established growth temperature of 24 °C, the effect of different sucrose concentrations on callus cultures was investigated. As reported in Figure 1b, with an increasing sucrose concentration, an increase of biomass productivity was observed, reaching the highest value of 0.016 g DW day^−1^ at 30 g L^−1^ of sucrose after 21 days. Lastly, different inoculum sizes for cellular suspensions of *C. cardunculus* were tested (Figure 1c). After 10 days of cultivation, about 1 g DW L^−1^ day^−1^ of biomass productivity was achieved for cellular suspensions with inoculum sizes of 100 and 150 g (fresh weight) L^−1^. With 50 and 200 g FW L^−1^ instead, the lowest biomass productivity was observed.

### 2.2. Utilization of Organic Carbon Sources by Cynara cardunculus Cellular Suspensions

**Figure 2:**
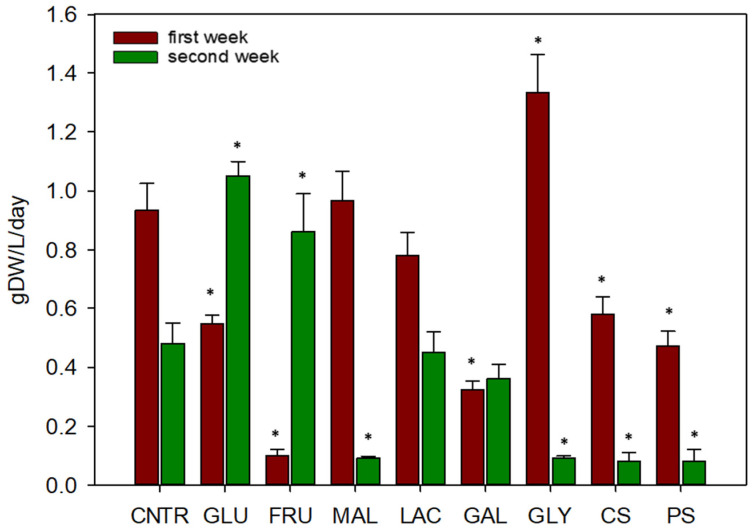
Comparison of biomass productivity for *C. cardunculus* cellular suspensions on different carbon sources after the first and the second week of cultivation. Asterisks (*) show the statistical difference between control and treatments. (CNTR is sucrose, GLU is glucose, FRU is fructose, MAL is maltose, LAC is lactose, GAL is galactose, GLY is glycerol, CS is corn starch, PS is potato starch.).

Biomass productivity of cellular suspensions, after 7 days and 14 days of cultivation, was reported in Figure 2. In particular, biomass productivity at the second week was calculated at 14th day with respect to the 7th day.

After one week, glycerol resulted as the best organic carbon source for cardoon cells growth, showing a significant different biomass productivity (1.33 g DW L^−1^ day^−1^) with respect to the control with sucrose (0.93 g DW L^−1^ day^−1^). Other carbon sources, such as glucose, fructose, galactose, and starches, instead, showed a significant lower productivity at 7 days. The presence of fructose and glucose in growth media determined a greater biomass productivity during the second week rather than the first one. In particular, during the second week a higher biomass productivity, statistically different than the control (0.48 g DW L^−1^ day^−1^), was observed for fructose (0.86 g DW L^−1^ day^−1^) and glucose (1.05 g DW L^−1^ day^−1^). A decrease of biomass productivity, instead, was observed for maltose, glycerol, and starches during the second week. These data resulted statistically different with respect to the control. Finally, only in presence of galactose the biomass productivity resulted stationary during the first and second week of cultivation.

### 2.3. Chemical Analysis

#### 2.3.1. Fatty Acids Analysis

**Figure 3:**
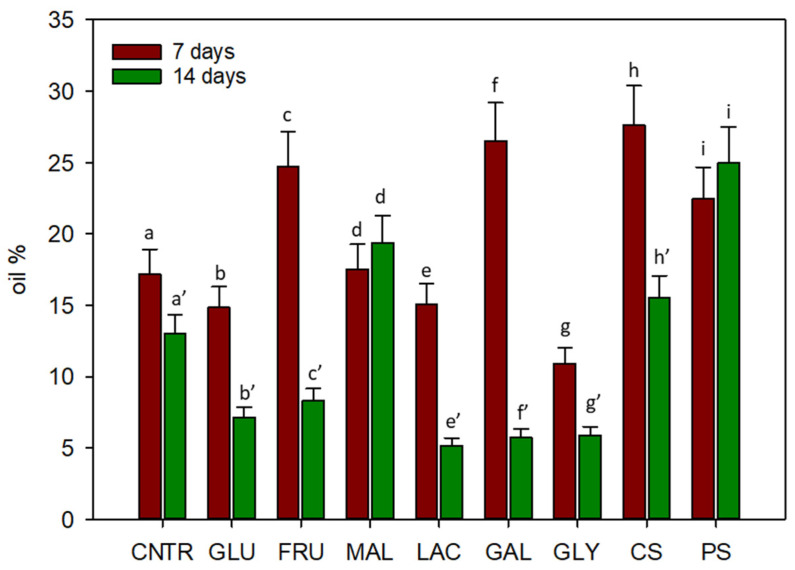
Percent oil content in *C. cardunculus* cellular suspensions grown on different carbon sources after 7 and 14 days of cultivation. (CNTR is sucrose, GLU is glucose, FRU is fructose, MAL is maltose, LAC is lactose, GAL is galactose, GLY is glycerol, CS is corn starch, PS is potato starch). Different letters indicate significant differences (*p* < 0.05) using the Duncan’s multiple range test.

*C. cardunculus* cellular suspensions, grown on different organic carbon sources for 7 and 14 days, were collected and lipid content was evaluated (Figure 3).

After 7 days of cultivation, *C. cardunculus* cells on sucrose showed a percentage of lipids equal to 17.21%; while the highest lipid content was observed when the media were added with corn starch (27.6%), galactose (26.5%), and fructose (24.7%). After 14 days of cultivation, the highest content in oil was reached in presence of maltose (19.4%) and potato starch (25%). All other organic carbon sources, instead, determined a low percentage of lipids (6.5%, on average) with respect to the control (13%) in the second week.

The fatty acids profile, reported in Table 1, showed that at the end of cultivation the most abundant fatty acids found in *C. cardunculus* suspensions were palmitic, linoleic, stearic, oleic, and linolenic.

Palmitic oil represents the largest fraction of oil. After 14 days of cultivation, cardoon cells grown with maltose, showed a higher percentage of palmitic (52.1%) and stearic (21.5%) oils with respect to the control on sucrose (46.2% of palmitic and 7.6% of stearic). High content of oleic oil, instead, was detected in cells grown on glycerol, starches, fructose, and glucose. On these carbon sources, in fact, the percentage of oleic oil resulted, on average, the double (14.1%) with respect to the control (6.7%).

#### 2.3.2. Polyphenols Analysis

**Figure 4:**
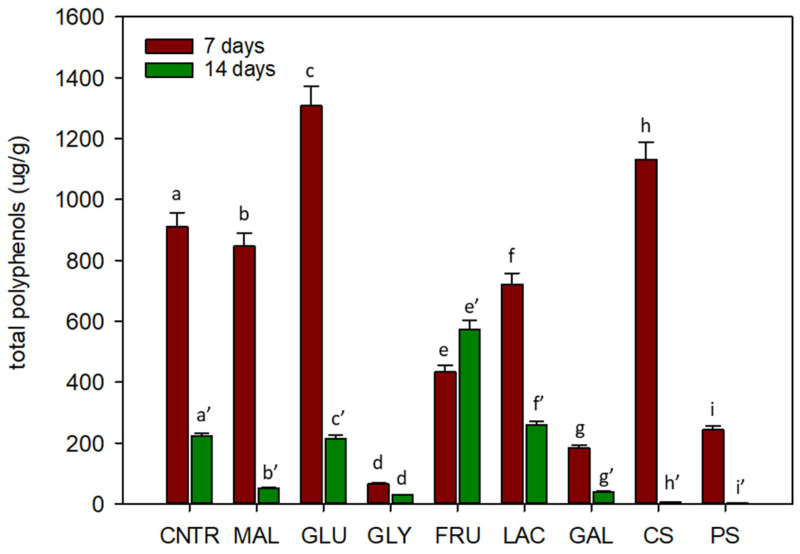
Content of total polyphenols, expressed as µg g^−1^ of dry weight, in *C. cardunculus* cellular suspensions after 7 and 14 days of cultivation on different carbon sources. (CNTR is sucrose, GLU is glucose, FRU is fructose, MAL is maltose, LAC is lactose, GAL is galactose, GLY is glycerol, CS is corn starch, PS is potato starch.). Different letters indicate significant differences (*p* < 0.05) using the Duncan’s multiple range test.

The analysis of total polyphenols showed that a high content of polyphenols was detected after one week of cultivation (Figure 4).

After 7 days, the highest content in polyphenols was observed in cardoon cells grown on glucose (1307.6 µg g^−1^) and corn starch (1131.6 µg g^−1^), with respect to the control (911 µg g^−1^). Generally, after 14 days of cultivation, polyphenols content decreased, and the highest value was reported for fructose (573.3 µg g^−1^).

Polyphenols found in *C. cardunculus* cells grown with different carbon sources are reported in Table 2.

The phenolic compounds in *C. cardunculus* biomass extracts were identified by matching their molecular ions (*m*/*z*) obtained by ESI-MS and ESI-MS/MS chromatographic characteristics with the literature data reported [22,23] or with available reference standards. In Table 3 the identified compounds, their retention time, and exact mass spectra data were reported, while the relative HPLC chromatogram was reported in Supplementary Materials (Figure S1). The data showed that the main polyphenols were 1,5-DiCQA and chlorogenic acid. In particular, cardoon cells on medium added with fructose showed, in absolute, the highest content in 1,5-DiCQA (332.8 µg g^−1^) and in chlorogenic acid (175.8 µg g^−1^). High content of 1,5-DiCQA and chlorogenic acid was also observed for cells grown on lactose (159.8 and 61.7 µg g^−1^ respectively).

## 3. Discussion

The capability of a specific carbon source to support plant cells growth depends on plant species and tissues. In particular, the inability of cells to use a specific carbon source may be due to the lack of the necessary enzymes; or to the inability to uptake the sugar due to specific impermeability [24].

Before assessing the capability to use different organic carbon sources in *C. cardunculus* cells, an optimization of their growth conditions was performed. The results showed that the best temperature and sucrose concentrations were 24 °C and 30 g L^−1^ respectively. Actually, as reported in literature, these values represented the growth conditions normally used for the cultivation of *C. cardunculus* cellular suspensions [25,26]. About the inoculum size, our results showed that increasing the inoculum from 50 g (fresh weight) L^−1^ to 150 g (fresh weight) L^−1^, the biomass productivity increased. In fact, it is reported that the larger the inoculum size, the earlier the stationary phase is reached [27,28]. However, above the value of 150 g (fresh weight) L^−1^ a decrease of growth was observed, probably due to the evidence that, in plant suspensions, high cell density could reduce oxygen transfer efficiency [29].

Once established the optimal growth parameters, eight organic carbon sources were tested on cardoon cells and, the best carbon source after the first week of cultivation was glycerol. In literature it is reported that exogenous glycerol could have negative effects on plant growth, such as inhibition and modifications in root development process [30]. However, in agreement with our results, Ben-Hayyim and Neumann [31] showed that 25 g L^−1^ of glycerol can be used as a carbon source for supporting the growth of *Citrus* callus.

The media with maltose and lactose, instead, induced a biomass productivity comparable to media with sucrose after 7 days of cultivation. Maltose can be rapidly accumulated and transported by leaves and plant roots playing an important role as osmoprotectant. In fact, this sugar can induce water stress tolerance in plants [32]. Moreover, Limberg et al. [24] isolated two variants from a suspension culture of soybean, able to grow rapidly on maltose as sole organic carbon source. About the use of lactose, instead, different results could be found in the literature, probably because it behaves differently depending on the species considered. In *Medicago sativa*, an increase of intracellular lactase activity was observed in cellular suspensions growing on lactose, probably due to an induction of enzyme synthesis; while cell-wall β-galactosidase seems not to be involved in lactose utilization [33]. Differently, in cucumber cell cultures, the presence of lactose in the medium induced an increase in extracellular β-galactosidase activity and lactose utilization starts only after a long lag phase [34]. However, a recent study, reported that *Rubus arcticus* cells showed a biomass reduction when lactose was used in substitution of sucrose [21].

Other organic carbon sources, like glucose and fructose, started to be used by *C. cardunculus* cells during the second week of cultivation. Plant cells respond differently to fructose and glucose. Sucrose was rapidly converted extra-cellularly into glucose and fructose, but glucose was uptaken preferentially [35]. Actually, our results showed that cardoon cells are able to use glucose better than fructose during the first week of cultivation. After that, fructose begins to be metabolized by the cells. This reduced uptake of fructose, compared to glucose, could in part be explained by the eight-fold lower affinity of the hexose carrier in the plasma membrane for fructose [35]. For example, when using a combination of glucose and fructose for the growth of *Phaseolus vulgaris* cell suspensions, the uptake of glucose is preferential. Nevertheless, when glucose and fructose are supplied individually, *P. vulgaris* cells used both efficiently [36].

Galactose and starches were the least used organic carbon sources by *C. cardunculus* cells. Galactose, in particular, has been considered toxic for many plants tissue cultures [31]. Actually, the negative effects of galactose on cellular growth are also observed by Cabasson et al. [37] which reported that galactose inhibits synthesis of UDP-glucose, an intermediate required for the cell wall synthesis.

*C. cardunculus* plants represent an important source of bioactive compounds. In fact, their in-field cultivation aims to obtain several metabolites that can find many applications in pharmaceutical and nutraceutical industry [4]. The main bioactive molecules are flavonoids (such as apigenin and luteolin), hydroxycinnamic derivatives (mono- and di-caffeoylquinic acids) and, in particular, polyphenols such as chlorogenic acid with important antioxidant functions [38]. Besides these compounds, *C. cardunculus* is an oleaginous species that produces seed oils which have been also investigated for human consumption [6]. Actually, this study evidenced polyphenols and oil production also from in vitro cardoon cultures. In particular, our results showed that the main polyphenols found in cardoon cells, cultivated on sucrose and alternative organic carbon sources, were 1,5-DiCQA and chlorogenic acid. Among the fatty acids, instead, the main constituent was palmitic acid. Polyphenols and fatty acids profiles found in in vitro cultures are comparable with those found in plant tissues of *C. cardunculus*. In fact, a comprehensive analysis of phenylpropanoids and fatty acids content in seeds, hypocotyls, cotyledons, and leaves for three different cardoon genotypes, evidenced that chlorogenic acid was the most produced polyphenol, reaching concentrations between 786.031 and 3735.790 µg g^−1^; while the most abundant fatty acid was represented by palmitic acid [38].

Based on chemical analysis, biomass productivity does not appear to be directly related to production of secondary metabolites and lipids. Therefore, a good carbon source for cellular suspension growth not necessarily is a good substrate for the production of high value-added compounds [16]. Our results showed that fructose, galactose, and starches induced low biomass productivity and high lipid production in cardoon cells after 7 days of cultivation. Regarding polyphenols production, a similar situation could be evidenced: glucose and corn starch determined low biomass productivity during the first week, with respect to sucrose, but induced a high production of polyphenols, in particular 1,5-dicaffeoylquinic acid. During the second week instead, fructose represents the best source of 1,5-dicaffeoylquinic acid. These results could be explained based on the evidence that, in plants, the phenolic content and the activity of enzymes of phenolic metabolism increase in response to environmental stress [26]. In particular, in stress conditions, the decrease of biomass growth could be due to an increased flux of carbon skeletons into the metabolic pathway of phenolic compounds [26,39].

The production of secondary metabolites depends also on the amount and type of carbohydrates in the growth medium. Gertlowski and Petersen [19] investigated the effects of carbon sources on rosmarinic acid production in cellular suspensions of *Coleus blumei*. The authors showed that when cellular growth becomes limited by depletion of other nutrients, the residual sugar in the medium influenced the amount of rosmarinic acid synthesized by the cell cultures: the more carbon is left in the medium the higher is the amount of rosmarinic acid produced.

In conclusion, this study provided evidence that alternative organic carbon sources, other than sucrose, could be used for in vitro cultivation of *C. cardunculus* cells. In particular, during the first week of cultivation, when sucrose is replaced by maltose, lactose, and glycerol (used separately), these provide the carbon needed for the growth. In the second week instead, also sugars like fructose started to be used by cardoon cells. Nevertheless, chemical analysis highlighted that carbon source inducing an optimal biomass productivity, not necessarily also induces an optimal productivity in terms of high value-added molecules. These findings lay the foundations for future investigations on the potential use of industrial streams as organic carbon source for the cultivation of plant cells, also in order to produce metabolites of industrial interest with a view to environmental sustainability.

## 4. Materials and Methods

### 4.1. In Vitro Cell Cultures of Cynara cardunculus: Optimization of Growth Conditions

To establish the optimal growth conditions to be used in subsequent cell growth tests, a previous evaluation of effects of temperature, sucrose concentrations, and inoculum size, was performed on in vitro cell cultures of *C. cardunculus*. In particular, the optimization of growth conditions was carried out by three consecutive steps: 1) Three different temperatures (20, 24, and 28 ± 1 °C) were tested on callus cultures to find the optimal value of temperature; 2) three different sucrose concentrations (10, 20, and 30 g L^−^^1^) were tested on callus cultures to find the optimal concentration of sucrose; 3) four different inoculum sizes (50, 100, 150, and 200 g of fresh weight L^−^^1^), intended as starting callus concentration for the preparation of cell suspensions, were tested.

To test different temperatures, *C. cardunculus* calluses were exposed to 20, 24, and 28 ± 1 °C using a thermostatic chamber. Nine Petri dishes, each containing five pieces of callus on agar Gamborg B5 (GB5) at 3% of sucrose, were prepared for each treatment and control. The initial callus content for each Petri dish was 1 ± 0.2 g. At each evaluation time (i.e., 7, 14, and 21 days) three Petri dishes were collected to determine the growth in terms of dry weight (DW): callus pieces were dried at 60 °C overnight and weighed. The growth of callus cultures, at 7, 14, and 21 days, was expressed as biomass productivity calculated following the Formula (1):

Biomass Productivity (gDW day^−1^) = (FW−IW)/T (1)

where:

FW is the final weight of calluses,

IW is the initial weight of calluses,

T is the exposure time.

After establishing the optimal growth temperature, different sucrose concentrations were tested: the test was carried out on callus cultures as described above and after 21 days the growth was calculated in terms of biomass productivity following the Formula (1).

Lastly, inoculum size was also investigated: *C. cardunculus* cell suspensions were prepared starting from friable callus transferred to a 250 mL Erlenmeyer flask containing 150 mL of GB5 at 3% of sucrose [25]. Four different inoculum sizes were tested and, consequently, a different amount of friable callus was dissolved in the medium for each: 7.5, 15, 22.5, and 30 g (fresh weight) of friable callus were used in order to reach an inoculum size of 50, 100, 150, and 200 g (fresh weight) L^−^^1^, respectively. Three replicates for each treatment were made. Cell suspensions were incubated at 24 ± 1 °C on orbital shaker (100 rpm) in the dark. Cellular growth was evaluated at 3, 7, and 10 days in terms of biomass productivity: 10 g (fresh weight) of cell suspension was dried at 60 °C overnight and expressed as cellular concentration (gDW L^−^^1^); then, biomass productivity for cellular suspension was calculated following the Formula (2):

Biomass Productivity (gDW L^−1^ day^−1^) = (Cf-Ci)/T (2)

where:

Cf is the final cellular concentration,

Ci is the initial cellular concentration,

T is the incubation time.

### 4.2. Cynara cardunculus Cellular Suspensions: Growth Test on Organic Carbon Sources

Cardoon cells growth was tested in presence of eight different organic carbon sources: galactose (GAL), maltose (MAL), glucose (GLU), glycerol (GLY), fructose (FRU), lactose (LAC), corn starch (CS), and potato starch (PS). Each chemical was added to GB5 standard medium at 30 g L^−^^1^, in substitution to sucrose. A positive control (CNTR) with sucrose (30 g L^−^^1^) was also performed. In detail, carbon content for each material was: 11.7 g L^−^^1^ for glycerol; 12 g L^−^^1^ for galactose, glucose, and fructose; 12.6 g L^−^^1^ for sucrose, lactose, and maltose; 12.9 g L^−^^1^ for starch. The test was carried out in Linfaboxes (Micropoli) by inoculating cardoon cells in 100 mL of GB5 added with each sugar, in order to reach a final cellular concentration of 150 g L^−^^1^ (fresh weight). The cellular suspension used as inoculum was allowed to settle in order to remove the residual medium. Then, the suspensions were incubated at 24 ± 1 °C on orbital shaker (100 rpm), in the dark, for 14 days. Three replicates were made for each treatment. Cellular growth, at 7 and 14 days, was expressed as biomass productivity and calculated as reported in Equation (2).

### 4.3. Chemical Analysis

#### 4.3.1. Lipid Extraction

The oil from the samples was extracted using solvent extraction as reported in Paolo et al. [40]. Briefly, lipids were extracted from 10 g of freeze-dried sample using 100 mL of hexane shaking the mixture for 24 h in a conical flask. Then the solution was filtered through a filter paper (Whatman No. 1) under reduced pressure and the hexane was completely evaporated by a rotary evaporator (Rotavapor RE 120; Büchi, Flavil, Sweden). The final weight of the extract oil after evaporation was recorded and used for the fatty acid analysis.

#### 4.3.2. Fatty Acids Analysis

Fatty acid analysis was performed by GC/MS (Agilent Technologies, Santa Clara, CA, USA). All sample were separated through a capillary column (Rxi-5MS 5% Phenyl 95% Dimethylpolysiloxane, Restek, Bellefonte, PA, USA). Helium was used as the carrier gas with a flow rate of 1.4 mL/min. The temperature of the injector was 280 °C. Aliquots (1 µL) were injected in splitless mode. Fatty acid methyl ester derivatives (FAMEs) were separated with the following temperature program: (1) 50 °C for 1.5 min; (2) increase to 80 °C at 2.5 °C/min for 1 min; and (3) increase to 300 °C at 10 °C/min with a total run time of 36 min. The row data were processed by the ChromaTOF software version 5.03.09.0 (LECO Corporation, Saint Joseph, MI, USA) that performs peak identification using the NIST11 library (NIST, MD, USA) after deconvolution of mass spectra. Results were expressed in relative percentages as mass response areas.

#### 4.3.3. Extraction of Polyphenols

The ultrasound-assisted extraction was performed as reported in our previous work [38]. Three grams of the freeze-dried sample was extracted with 30 mL of ethanol/water (50:50 *v*/*v*) by sonication at room temperature for 30 min, centrifuged at 4 °C (10 min, 4000× g), filtered through 0.45 µm nylon membranes, and then used for high-resolution mass spectrometry and total polyphenolic content analyses. The ultrasonic plant material extraction procedure was repeated three times.

#### 4.3.4. UHPLC-HRMS Analysis of Polyphenols

The qualitative and quantitative analysis of polyphenols extracted from cardoon cell cultures was performed via high-resolution mass-spectrometry using an ultra-high pressure liquid chromatograph Dionex UltiMate 3000 (UHPLC, Thermo Fisher Scientific), coupled with a Q-Exactive Orbitrap mass spectrometer (Thermo Fisher Scientific). A UHPLC system (UHPLC, Thermo Fisher Scientific, Waltham, MA, USA) was used for the quantification and separation of polyphenolic compounds. The details of UHPLC-high-resolution mass spectrometry analysis are as described by Graziani et al. [38].

#### 4.3.5. Total Polyphenolic Content Determination

Polyphenol contents were determined using Folin–Ciocalteau assay as reported by Box et al. [41]. Few modifications have been made, in particular, 125 μL of polyphenolic extract (diluted 10 times) was mixed with 500 μL of deionized water and 125 μL of the Folin–Ciocalteu reagent for 6 min at room temperature. Then, 1 mL of deionized water and 1.25 mL of 7.5% of sodium carbonate solution were added to the mixture. After 90 min of incubation in the dark, the absorbance at 760 nm was measured. Concentrations of total phenolic were expressed as mg of gallic acid equivalents (GAE) per gram of dry weight (DW). Each extract was analyzed in triplicate.

### 4.4. Statistical Analysis

All data are reported as mean ± SD. One-way ANOVA was applied to test for significant differences between treatments and control (significance level was always set at *p* = 0.05). The data were analyzed using IBM^©^ SPSS^©^ Statistics software Ver. 23 (SPSS, Inc., Chicago, IL, USA).

## Figures and Tables

**Table 1:** Fatty acids composition, expressed as % of the total fatty acid composition, in *C. cardunculus* cells after 14 days of cultivation on different organic carbon sources. Each value represents the mean of three replicates. Different superscript letters denote a significant difference between organic carbon sources by analysis of variance [ANOVA]. Statistical significance was defined as *p* < 0.05, using Tukey’s post hoc test for mean separation. Data are reported as mean ± SD. n.d.: not detected.

	CNTR	MAL	GLU	GLY	FRU	LAC	GAL	CS	PS
Palmitic (C16:0)	46.2 ± 0.6 ^a^	52.1 ± 0.2 ^b^	38.9 ± 0.2 ^c^	47.8 ± 3.1	40 ± 0.5 ^d^	49.1 ± 0.2	40.8 ± 0.3 ^d^	45.1 ± 0.1 ^a^	36.4 ± 0.7 ^e^
Stearic (C18:0)	7.6 ± 0.1 ^a^	21.5 ± 0.2 ^b^	8.4 ± 0.1 ^c^	12.2 ± 0.8 ^d^	9.5 ± 0.1 ^e^	13 ± 0.1 ^d^	21.3 ± 0.1 ^b^	29.8 ± 0.1 ^f^	15.5 ± 0.3 ^g^
Oleic (C18:1)	6.7 ± 1.8 ^a^	1.5 ± 0.1 ^b^	12.3 ± 0.8 ^c^	17 ± 5.4 ^d^	12.6 ± 1.2 ^c^	4 ± 1.3 ^e^	11.3 ± 0.1 ^f^	12.2 ± 0.2 ^c^	16.3 ± 1.1 ^g^
Linoleic (C18:2)	27.3 ± 0.8 ^a^	16.9 ± 0.1 ^b^	29.5 ± 0.4 ^c^	21.7 ± 1.3 ^d^	27.3 ± 0.4 ^a^	21.8 ± 0.5 ^d^	19.9 ± 0.1 ^e^	11.0 ± 0.1 ^f^	31.1 ± 0.2 ^c^
Linolenic (C18:3)	9.8 ± 0.2 ^a^	6.9 ± 0.2 ^b^	9 ± 0.1 ^c^	n.d.	9.2 ± 0.2 ^c^	9.9 ± 0.6 ^a^	5.4 ± 0.3 ^d^	n.d.	n.d.
Others	2.4 ± 0.3 ^a^	0.9 ± 0.1 ^b^	1.9 ± 0.1 ^c^	1.3 ± 0.2 ^d^	1.3 ± 0.2 ^d^	2.1 ± 0.2 ^c^	1.2 ± 0.1 ^b^	0.9 ± 0.1 ^b^	0.6 ± 0.2 ^e^

**Table 2:** Polyphenols content, expressed as µg g^−1^ of dry weight, in *C. cardunculus* cells after 14 days of cultivation. Data are reported as mean ± SD. Different superscript letters denote a significant difference between different organic carbon sources by analysis of variance [ANOVA]. Statistical significance was defined as *p* < 0.05, using Tukey’s post hoc test for mean separation. n.d.: not detected.

	Peak ID	CNTR	MAL	GLU	GLY	FRU	LAC	GAL	CS	PS
3-CQA	1	44.1 ± 3.1 ^a^	5.3 ± 0.4 ^b^	39.6 ± 3.2 ^c^	2.1 ± 0.1 ^d^	175.8 ± 10.5 ^e^	61.9 ± 4.9 ^f^	2.9 ± 0.2 ^d^	0.8 ± 0.1 ^g^	0.4 ± 0.1 ^h^
*p*-Coumaric acid	2	7 ± 0.5 ^a^	2.8 ± 0.2 ^b^	4.6 ± 0.4 ^c^	1.6 ± 0.1 ^d^	7.1 ± 0.5 ^a^	4.4 ± 0.4 ^c^	2.2 ± 0.2 ^d^	0.7 ± 0.1 ^e^	0.7 ± 0.1 ^e^
3-FQA	3	6.4 ± 0.5 ^a^	6.3 ± 0.4 ^a^	7.1 ± 0.3 ^b^	7.8 ± 0.5 ^c^	5.6 ± 0.4 ^d^	8.4 ± 0.7 ^e^	3.3 ± 0.2 ^f^	n.d.	n.d.
Ferulic acid	4	6.6 ± 0.5 ^a^	1.3 ± 0.1 ^b^	7.1 ± 0.6 ^c^	1.7 ± 0.1 ^d^	16.1 ± 0.8 ^e^	2.7 ± 0.2 ^f^	1.8 ± 0.1 ^d^	0.5 ± 0.1 ^g^	0.3 ± 0.1 ^h^
5-FQA	5	9.5 ± 0.7 ^a^	2.6 ± 0.2 ^b^	7.1 ± 0.6 ^c^	2.9 ± 0.2 ^d^	15.6 ± 1.1 ^e^	12.4 ± 0.7 ^f^	3.3 ± 0.2 ^g^	n.d.	n.d.
3,4-DiCQA	6	9.1 ± 0.5 ^a^	5.4 ± 0.4 ^b^	20.5 ± 1.4 ^c^	7.3 ± 0.4 ^d^	17.8 ± 0.9 ^e^	8 ± 0.6 ^f^	15.6 ± 0.9 ^g^	0.5 ± 0.1 ^h^	0.2 ± 0.1 ^i^
1,5-DiCQA	7	137.4 ± 8.2 ^a^	25.3 ± 2 ^b^	128 ± 7.7 ^c^	6.4 ± 0.4 ^d^	332.8 ± 23.3 ^e^	159.8 ± 12.8 ^f^	9.9 ± 0.9 ^g^	1.7 ± 0.1 ^h^	1.1 ± 0.1 ^i^
5-iFQA	8	0.4 ± 0.1 ^a^	0.2 ± 0.1 ^b^	0.4 ± 0.1 ^a^	0.2 ± 0.1 ^b^	2.4 ± 0.2 ^c^	0.7 ± 0.1 ^d^	n.d.	0.2 ± 0.1 ^b^	n.d.

**Table 3:** Retention time and exact mass spectra data of phenolic compounds investigated by UHPLC-HRMS Orbitrap. RT = retention time.

Compounds	RT	Molecular Formula	Theoretical Mass [M-H]^−^	Experimental Mass [M-H]^−^	MS/MS Ions	Accuracy (Δppm)
3-CQA	8.30	C_16_H_17_O_9_	353.08798	353.08783	191.05571–179.03461	−0.42
*p*-Coumaric acid	9.58	C_9_H_8_O_3_	163.03917	163.03912	119.04981	−0.31
3-FQA	9.75	C_17_H_19_O_9_	367.10345	367.10335	193.04961	−0.27
Ferulic acid	9.88	C_10_H_10_O_4_	193.05063	193.05016	179.05556	−2.43
5-FQA	10.82	C_17_H_19_O_9_	367.10345	367.10315	191.05501	−0.82
3,4-DiCQA	11.21	C_25_H_23_O_12_	515.11950	515.11969	353.08755–191.05561	0.37
1,5-DiCQA	11.64	C_25_H_23_O_12_	515.11950	515.11957	353.08753–191.05550	0.14
5-iFQA	11.86	C_17_H_19_O_9_	367.10345	367.10309	193.04915	−0.98

## Data Availability

Not applicable.

## Supplementary Materials

The following supporting information can be downloaded at: www.mdpi.com/article/10.3390/plants11050701/s1, Figure S1: HPLC chromatogram for phenolic compounds.
