# Supplementary material for: Use of Different Organic Carbon Sources in Cynara cardunculus Cells: Effects on Biomass Productivity and Secondary Metabolites
Source: Plants (Basel). 2022 Mar 5;11(5):701. doi: 10.3390/plants11050701 (PMC8912832; doi:10.3390/plants11050701)
Supplement: Supplementary file 1 [file plants-11-00701-s001.zip › plants-1596299-supplementary.pdf]

Supplementary materials.

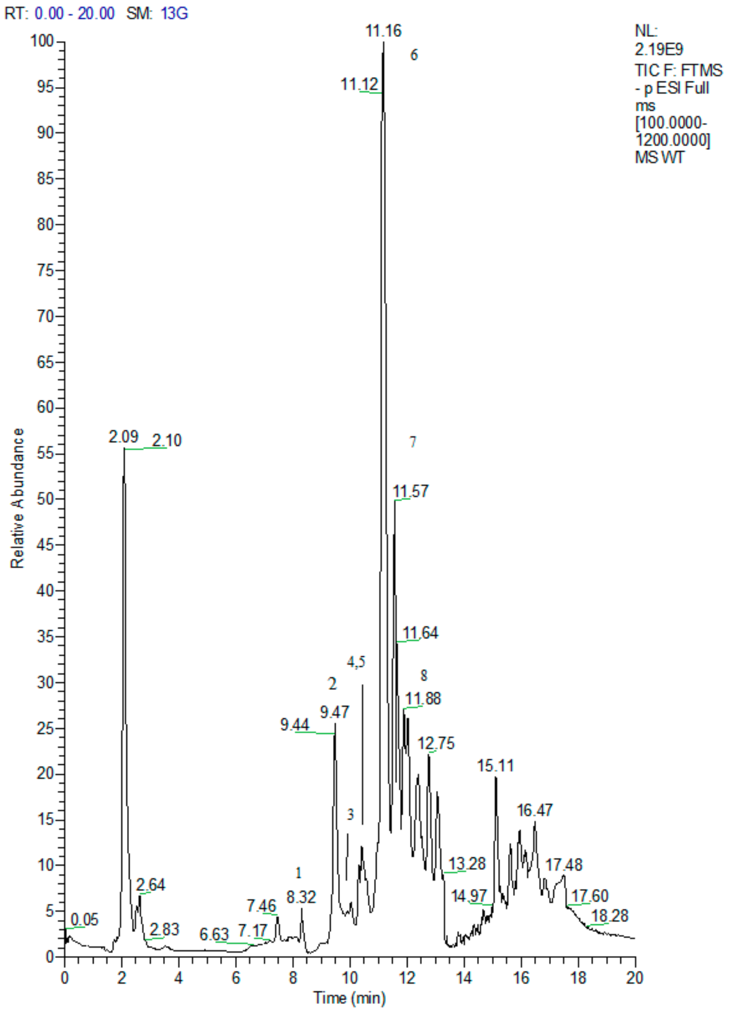

Figures S1. HPLC chromatogram for phenolic compounds. Peak numbers refer to phenolic compounds are reported in Table 2.
